# Colocated MIMO Radar Waveform-Array Joint Optimization for Sparse Array

**DOI:** 10.3390/s23094375

**Published:** 2023-04-28

**Authors:** Jinrong Yin, Rui Ma, Mingcong Lin, Shenghua Zhou

**Affiliations:** 1 Nanjing Institute of Electronic Technology, Najing 210039, China; 2 National Laboratory of Radar Signal Processing, Xidian University, Xi’an 710071, China

**Keywords:** colocated MIMO radar, waveform optimization, array optimization, sparse array, range sidelobe suppression

## Abstract

Colocated multiple-input multiple-output (MIMO) radar can transmit a group of distinct waveforms via its colocated transmit antennas and the waveform diversity leads to several advantages in contrast to conventional phased-array radar. The performance depends highly on the degrees available, and element spacing can be deemed as another source of degrees of freedom. In this paper, we study the joint waveform and element spacing optimization problem. A joint waveform and array optimization criterion is proposed to match the transmit beampattern, the suppression range, and the angular sidelobes, under the constraints of minimal element spacing and total array aperture. Meanwhile, the effect of receive beamforming on suppressing mutual correlation between returns from different spatial directions is also incorporated into the optimization criterion. The optimization problem is solved by the sequential quadratic programming algorithm. Numerical results indicate that with more degrees of freedom from array spacings, colocated MIMO radar achieves a better transmit beampattern matching performance and a lower sidelobe level, compared with a fixed half-wavelength spaced array, but the benefits from additional degrees of freedom from array spacing optimization have a limit.

## 1. Introduction

Space-borne radar can search for targets in a greater volume from space and thus always receives intensive attention from researchers in many countries. Unlike radar systems on other platforms, space-borne radar systems [1] put higher standards on stability, robustness, and survivability in space. At the end of the 20th century, techniques regarding space-borne radar grew rapidly, and smart satellites provided another solution for space-borne radar. Just like unmanned aircrafts, such smaller satellites may fly together stably in space; they could be considered distributed antennas of a novel radar system with high stability, robustness, and survivability.

The antenna array may operate in the developed phased-array radar mode, but now multiple-input multiple-output (MIMO) radar [2], with more degrees of freedom and better performance in many aspects, is a better choice. According to the distance between radar antennas, MIMO radar can be classified into two kinds, i.e., distributed MIMO radar [3] and colocated MIMO radar [4]. Both kinds of MIMO radar have several advantages over their conventional counterparts [4,5]. The former has widely separated radar antennas to observe different aspects of radar targets, whereas the latter has colocated antennas in space to observe only one aspect of targets. The criterion to determine whether two signals are received by two diversity channels can be found in [6]. The distributed MIMO radar is generally incoherent, i.e., the phase differences between transmit/receive antennas are either not coordinated or not exactly known, because with widely separated radar antennas, independent target returns and independent interference are often obtained and the optimal processing algorithms are incoherent in most situations. The colocated MIMO radar is coherent on both the transmit and the receive ends and can operate in a much more flexible mode than phased-array radar. The coherence between antennas can achieve a much longer detection distance with numerous coherent antennas; thus, this type of radar is more suitable for space-borne radar to detect targets at a long distance.

Well-designed waveforms are critical to realize claimed advantages, and therefore, radar waveform optimization is a hot topic in the MIMO radar field [7,8,9,10]. For distributed MIMO radar, waveform optimization just needs to suppress auto and mutual correlation sidelobes of transmit waveforms [4] and thus is less sophisticated than that for colocated MIMO radar [11]. For colocated MIMO radar, however, waveform optimization can make colocated MIMO radar operate in a complicated mode, e.g., to steer multiple transmit beams one at a time into multiple spatial directions [12] (Phased-array radar can also illuminate multiple beams into multiple directions within one transmission, but the interference performance is worse than that for colocated MIMO radar). Therefore, the MIMO radar scheme may be an interesting choice for space-borne radar.

Waveform optimization for colocated MIMO radar mainly has two goals, i.e., matching a desirable transmit beampattern [4] and suppressing auto and cross-range sidelobes [10,11]. These two goals are often expressed in different forms. First, two different pursuits should be combined together in optimization and thus a trade-off is required. Second, it is difficult to match a directional transmit beampattern together with range sidelobe suppression. Third, there are different measures of the sidelobe level in existence, so nearly orthogonal waveforms designed for distributed MIMO radar are unsuitable to colocated MIMO radar even with an omnidirectional transmit beampattern because their sidelobe level measures are different [13].

If one ignores range sidelobes and concentrates on the transmit beampattern, the optimization problem may be convex, and then a global optimal point may be found [12]. A major difficulty for radar waveform optimization lies in range sidelobe suppression with the constant-modulus constraint, which comes from the fact that radar transmit circuits often operate in saturation mode. The saturation operation mode can circumvent the demand for accurate inner-pulse power control required by amplitude-modulated waveforms. For waveform optimization, however, the constant-modulus constraint would make range sidelobe suppression suffer from numerous local minima to reach the global optimal point. Therefore, we have to use optimization algorithms such as the genetic algorithm [9], simulated annealing algorithm [14,15], and sequential quadratic programming (SQP) algorithm [16,17]. For such optimization algorithms, the final performance relies on a subtle optimization function, but sufficient degrees of freedom are also critical. Rich degrees of freedom from signal diversity are the source of advantages of colocated MIMO radar and the key to yield better flexibility compared with its phased-array counterpart. However, in waveform optimization with range sidelobe suppression, the degrees of freedom are still insufficient in some situations. Smart antenna swarms in space can set the element spacing more flexibly and thus the element spacing can be considered as another kind of degrees of freedom for optimization.

In this paper, we consider waveform design for colocated MIMO radar with a sparse transmit array in the background of space-borne radar. The spacing of elements is optimized together with transmit waveforms. Meanwhile, an attenuation factor is introduced to measure how much the receive beamforming would affect cross-correlation sidelobes in spatial receive channels and then is incorporated into our waveform design criterion. We define three groups of quantized angular frequencies to match a transmit beampattern, to represent spatial receive channels, and to simulate target returns from various spatial directions. Unlike [12], who matches the transmit beampattern through the waveform covariance matrix, we directly squeeze the difference between the desirable transmit beampattern and real transmit beampattern. Meanwhile, an offline parameter is used to balance two pursuits and an offline parameter is used to control the total transmit aperture after optimization.

The sparse array in radar can achieve a large aperture with a given number of elements and the element positions should be optimized to avoid grating sidelobes [18]. For colocated MIMO radar, the sparsity of the transmit array can improve range resolution without introducing grating sidelobes in the receive end. That is different from phased-array radar, whose angular resolution is determined merely by the aperture of the receive array. Meanwhile, in a sparse array, the locations of elements may be optimized, which is also a source of degrees of freedom and has the potential to improve performance. In [19,20], transmit aperture optimization is addressed in the background of a low-cost lightweight antenna array and orthogonal waveforms are illuminated through antennas. In [21], a sparse circular array and its advantages are introduced. In [12], waveform covariance matrix optimization is addressed in transmit beampattern matching, but range sidelobes and array optimization are not addressed. In [22], waveforms for colocated MIMO radar have been optimized to enhance the anti-jamming performance, provided that an electronic attack device operates only in saturation mode, but the element spacing is not optimized for more degrees of freedom.

More performance improvement can be achieved by addressing the receive end processing. We notice that colocated MIMO radar needs to suppress sidelobes of angular waveforms [11], i.e., waveforms illuminated into different spatial directions, termed as angular waveforms in [23], and the receive beamforming operation [24] can make us impose slighter weights on suppressing cross-correlation of angular waveforms [16,25]. In nature, cross-correlation sidelobes of angular waveforms represent how much a spatial receive channel suffers from target returns from various spatial directions. In the receive end, the receive beamforming operation for array radar has the same function and is often more efficient in suppressing this kind of interference. Notwithstanding that, one has to suppress such cross-correlation sidelobes by waveform optimization [11], but the number of cross-correlation sidelobes is often greater than that of auto-correlation sidelobes; thus, many degrees of freedom are consumed on suppressing cross-correlation sidelobes. If we incorporate the effect of receive beamforming, less attention may be placed on cross-correlation sidelobes, and then some degrees of freedom can be released for better use. An extreme case is addressed in [16], where only auto-correlation sidelobes are suppressed through waveform optimization for a receive array with a larger aperture, leaving cross-correlation sidelobes suppressed at the receive beamforming stage. Huge sidelobe performance improvement is achieved.

Numerical results are given to show waveform optimization results. We find that additional degrees of freedom result in better sidelobe level and a better transmit beampattern performance, without grating sidelobes present in the receive end. Aided with receive beamforming, the overall sidelobe level reaches a much lower level. Meanwhile, a numerical simulation is also performed to examine the sidelobe level improvement, indicating that as the total aperture increases, the benefits have a limit.

The rest of this paper is organized as follows. In Section 2, the sidelobes and transmit beampattern of the sparse MIMO transmit array is formulated, the attenuation factor of the receive beamforming is introduced, and the waveform optimization criterion is presented. In Section 3, numerical results are given to show how much the additional degrees of freedom from spacing optimization affect transmit beampattern matching performance and the sidelobe level output. In Section 4, some discussions about parameter settings are given and some conclusions about their applications are drawn.

## 2. Waveform and Array Optimization for Sparse MIMO Array

**Figure 1:**
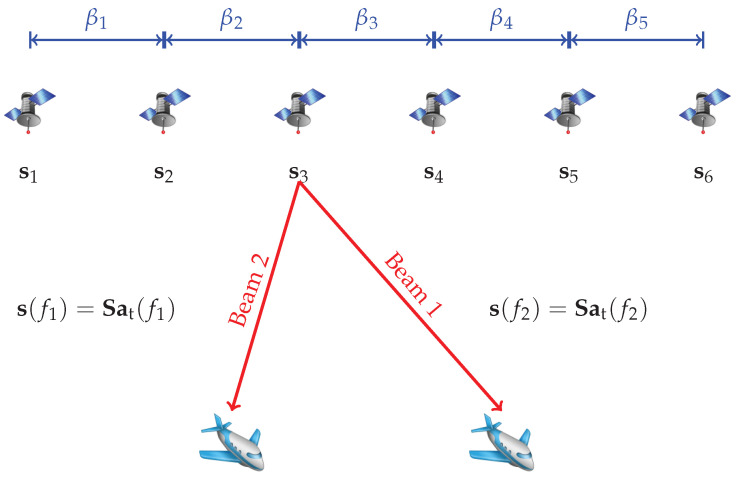
The system diagram of colocated MIMO radar array in space.

For simplicity, we consider a group of smart satellites flying in line at the same speed and carrying antennas in the same orientation. That is, a colocated MIMO radar system with a linear sparse $N_{t}$-element transmit array and a linear half-wavelength spaced receive array with $N_{r}$ elements. A system diagram is shown in Figure 1. Assume that the satellites can maneuver to construct a given distribution in space if needed; no position error is considered in this paper.

For the sparse transmit array, the distance between the *i*th element and the (i+1)th element is denoted by $d_{i}$, $i=1,2,\cdots ,N_{t}-1$. The transmit steering vector can then be written as

(1) $$a_{t}\left(\theta \right)={\left[1,\exp\left(j2\pi f_{0}d_{1}\sin\left(\theta \right)/c\right),\cdots ,\exp\left(j2\pi f_{0}\sum_{i=1}^{N_{t}-1}d_{i}\sin\left(\theta \right)/c\right)\right]}^{T}$$

where $f_{0}$ denotes carrier frequency, *j* denotes the imaginery symbol, exp(·) denotes the exponential function, θ is the spatial direction of interest, *c* is the speed of light, and ${\left[\cdot \right]}^{T}$ is the transpose operator.

To be concise, the directions are treated as a frequency term in the linear array configuration, and we define a normalized angular frequency by

(2) $$f_{c}=0.5\sin\left(\theta \right).$$

The element spacing between elements is normalized by the wavelength as

(3) $$\beta_{i}=2d_{i}/\lambda ,$$

where $\lambda =c/f_{0}$ denotes the wavelength. In particular, for a half-wavelength array, $d_{i}=\lambda /2$ for $i=1,\cdots ,N_{t}-1$.

With $\beta_{i}$, we can express the transmit steering vector in another form as

(4) $$a_{t}\left(f_{c}\right)=\exp\left(j,2,\pi ,L,\beta \right),$$

where the matrix

(5) $$L={\left[\begin{array}{ccccc} 0 & 0 & \cdots & 0 & 0\\ 1 & 0 & \ddots & \vdots & 0\\ \vdots & 1 & \ddots & 0 & \vdots \\ 1 & \vdots & \ddots & 0 & 0\\ 1 & 1 & \cdots & 1 & 0 \end{array}\right]}_{N_{t}\times \left(N_{t},-,1\right)}$$

translates element spacings to element positions, and $\beta ={\left[\beta_{1},\beta_{2},\cdots ,\beta_{N_{t}-1}\right]}^{T}$ is a vector of element spacings. For a uniform linear array, β is a vector of identical members. It is more convenient to run the optimization process over $\beta_{k}$ which all have the same range.

To control the overall aperture of the transmit array, we define the total aperture by

(6) $$D=\sum_{i=1}^{N_{t}-1}\beta_{i},$$

for which the real array aperture is Dλ/2.

In order to make a fair comparison with the aperture of a uniform half-wavelength spaced array, for which $D=N_{t}-1$, we define a measure of array aperture extension by

(7) $$\eta =D/\left(N_{t},-,1\right).$$

For the half-wavelength spaced linear array, η=1. As real antenna spacing often has a lower bound λ/2, we set η≥1, and it would increase with total aperture *D*.

### 2.1. Angular Waveform and Transmit Beampattern

Assume that the colocated MIMO radar system under concern has a transmit waveform matrix denoted by $S=[s_{1},s_{2},\cdots ,s_{N_{t}}]\in C^{N_{s}\times N_{t}}$, where $s_{i}$ is the waveform transmitted by the element on the *i*th satellite, and $N_{s}$ is the number of codes of each waveform.

After transmitted waveforms S are transmitted from transmit antennas into surveillance, they will constructively or destructively interfere with each other to form different waveform signatures in different spatial directions, subsequently termed as angular waveforms. For a spatial direction θ, a coherent combination of S would yield an angular waveform as

(8) $$s\left(f_{c}\right)=Sa_{t}\left(f_{c}\right)$$

The transmit beampattern is defined as the power of angular waveforms in different spatial directions, i.e.,

(9) $$\begin{array}{cc} p\left(f_{c}\right) & =s^{H}\left(f_{c}\right)s\left(f_{c}\right)/N_{s}\\ \; & =a_{t}^{H}\left(f_{c}\right)R_{0}a_{t}\left(f_{c}\right), \end{array}$$

where ${\left(\cdot \right)}^{H}$ denotes the conjugate transpose operator, and

(10) $$R_{0}=SS^{H}/N_{s}$$

denotes the transmit waveform covariance matrix of S.

### 2.2. Attenuation Factor of Receive Beamforming

We assume that the transmit waveforms are narrow-band. In this case, the waveform covering a target is an angular waveform and target returns also bear the same waveform signature, to which the receive end would match. There is often a Doppler modulation and we do not address this issue here. At the receive end, there are various signal processing algorithms, which differ mainly in the method of suppressing background interference [23]. In [24], several signal processing algorithms for colocated MIMO radar are proposed, for which the receive beamforming components all have the following form [26]:

(11) $$w_{r}\left(f_{c}\right)=\frac{R_{r}^{-1}a_{r}\left(f_{c}\right)}{a_{r}^{H}\left(f_{c}\right)R_{r}^{-1}a_{r}\left(f_{c}\right)},$$

where $R_{r}$ is an estimate of the interference covariance matrix and the receive steering vector is denoted by

(12) $$a_{r}\left(f_{c}\right)={\left[1,\exp\left(j2\pi f_{c}\right),\cdots ,\exp\left(j2\pi \left(N_{r}-1\right)f_{c}\right)\right]}^{T}.$$

The receive steering vector indicates that the receive array is a uniform array with a half-wavelength spacing.

Adaptive interference suppression algorithms, such as the MIMO–Capon algorithm [24], involve samples received online. Since interference circumstances may be different at different range cells and online waveform optimization involving range sidelobe suppression is difficult to implement, we do not concern ourselves with such adaptive algorithms at the current stage; rather, we formulate a simple and classical MIMO signal processing algorithm, i.e., the MIMO least square (LS) algorithm, for which $R_{r}=I$ and the receive beamforming weight is

(13) $$w_{r}\left(f_{c}\right)=a_{r}\left(f_{c}\right)/N_{r}.$$

In [23], the implementation of the MIMO LS algorithm is addressed; it is mainly composed of three operations, i.e., receive beamforming, range compression, and transmit synthesis. The latter two operations are realized by a concise unit called space-time range compressors, which follows a receive beamforming filter and is actually a matched filter regarding returns in directions associated with the beamforming filter. If a spatial receive channel regarding a spatial direction $f_{c}$ uses this receive beamforming weight, target returns from other spatial directions would be attenuated first by the beamforming filter before they pass the space-time range compressor. Cross-correlation sidelobes measure how they are attenuated in the space-time range compressor, whereas the precedent beamforming filter has attenuated them first. Therefore, a good combination of the suppression terms can make better use of the degrees of freedom.

It can be found that target return from $f_{c}^{\prime }$ is attenuated in the beamforming filter by a factor

(14) $$\rho_{r}\left(f_{c},f_{c}^{\prime }\right)=a_{r}^{H}\left(f_{c}^{\prime }\right)a_{r}\left(f_{c}\right)/N_{r}=a_{r}^{H}\left(f_{c}^{\prime }-f_{c}\right)1_{N_{r}}/N_{r},$$

which is termed as the attenuation factor.

In particular, if $f_{c}=f_{c}^{\prime }$ then $\rho_{r}=1$, standing for no attenuation; otherwise, $\rho_{r}$ is generally less than one and the value indicates the degree of attenuation. If $f_{c}$ deviates far from $f_{c}^{\prime }$, $\rho_{r}$ is generally very small. In this case, if receive beamforming can attenuate angular sidelobes efficiently, it is unnecessary to put too many degrees of freedom on mutual correlation sidelobe suppression in waveform optimization.

### 2.3. Sidelobes of Angular Waveforms

Notwithstanding target absolute amplitude, for target return $s(f_{c}^{\prime })$, the space-time range compressor intended to match angular waveform $s(f_{c})$ would output sidelobes given by

(15) $$\begin{array}{cc} \rho_{k}\left(f_{c},f_{c}^{\prime }\right) & =\frac{a_{t}^{H}\left(f_{c}\right)SJ_{k}S^{H}a_{t}\left({f_{c}}^{\prime }\right)}{a_{t}^{H}\left(f_{c}\right)SS^{H}a_{t}\left(f_{c}\right)}\\ \; & =\frac{1}{p\left(f_{c}\right)}a_{t}^{H}\left(f_{c}\right)R_{k}a_{t}\left({f_{c}}^{\prime }\right), \end{array}$$

where *k* denotes mutual range shift, and

(16) $$R_{k}=SJ_{k}S^{H}/N_{s}$$

denotes the shifted waveform covariance matrix. The shift matrix is defined by

(17) $$J_{k}=J_{-k}^{T}=\left[\begin{array}{cc} 0_{(N_{s}-k)\times k} & I_{N_{s}-k}\\ 0_{k\times k} & 0_{k\times (N_{s}-k)} \end{array}\right],$$

where 0 denotes an all-zero matrix with subscripts indicating its sizes, and I denotes the identity matrix.

In particular, if $f_{c}=f_{c}^{\prime }$, then we obtain auto-correlation sidelobes as

(18) $$\rho_{k}\left(f_{c}\right)=a_{t}^{H}\left(f_{c}\right)R_{k}a_{t}\left(f_{c}\right)/p\left(f_{c}\right).$$

From (17), we have

(19) $$R_{-k}=SJ_{-k}S^{H}/N_{s}={\mathrm{SJ}}_{k}^{T}S^{H}/N_{s}=R_{k}^{H},$$

and then

(20) $$\rho_{-k}\left(f_{c}\right)=\rho_{k}^{*}\left(f_{c}\right),$$

where ${\left(\cdot \right)}^{*}$ denotes the conjugate operator. In waveform design, it means that we can suppress only one side of range sidelobes, say, those for k>0.

Meanwhile, for cross correlation sidelobes, we have

(21) $$\;\rho_{-k}\left(f_{c},f_{c}^{\prime }\right)=\frac{p\left({f_{c}}^{\prime }\right)}{p\left(f_{c}\right)}\rho_{k}^{*}\left({f_{c}}^{\prime },f_{c}\right).$$

With this relationship, we can reduce the number of values to minimize as well.

### 2.4. Sidelobes after Range Compression

For a directional transmit beampattern with two peaks, the receive end generally deploys at least two space-time range compressors to deal with returns from those directions. Target returns from two directions would have nonzero outputs in both compressors and the mutual interference can be measured by cross-correlation sidelobes, which should thus be suppressed. As angular waveforms with two peaks also have conjugate symmetric sidelobes, we can suppress only one side of both auto- and cross-correlation sidelobes of two angular waveforms.

Such a range compressor may receive target returns from any spatial direction. Targets or clutter patches from other spatial directions may also have sufficient power to spoil the range compressors. In this case, we intend to suppress the outputs in the range compressors, but we need to suppress both sides of range sidelobes.

Here we focus on sidelobe outputs after receive beamforming and space-time range compression. In addition to the attenuation factor, the sidelobe outputs have a form as

(22) $${\bar{\rho }}_{-k}\left(f_{c},f_{c}^{\prime }\right)=\rho_{r}\left(f_{c},f_{c}^{\prime }\right)\times \rho_{-k}\left(f_{c},f_{c}^{\prime }\right).$$

As $\rho_{r}\left(f_{c},f_{c}\right)=1$, receive beamforming does not alter auto-correlation sidelobes.

### 2.5. Transmit Beampattern Matching

Transmit beampattern matching has mainly two approaches. One approach is to optimize a waveform covariance matrix that bears a certain beampattern; given such a waveform covariance matrix, transmit waveforms are optimized to match it [12]. Here we choose the other method, i.e., to directly squeeze the mismatch between the desirable transmit beampattern and real transmit beampattern. As the angular frequency is a continuous value, we need to quantize it first and then optimize it at selected angular frequencies.

Given a group of $N_{d}$ selected representative angular frequencies denoted by $f_{b}=\left[f_{b}\left(1\right),\cdots ,f_{b}\left(N_{b}\right)\right]$, we assume that desirable transmit beampattern responses are $b_{d}$. The real transmit beampattern at $f_{b}$ can be expressed as

(23) $$\begin{array}{cc} b_{R} & ={\left[p\left(f_{b}\left(1\right)\right),p\left(f_{b}\left(2\right)\right),\cdots ,p\left(f_{b}\left(N_{b}\right)\right)\right]}^{T}\\ \; & =\mathrm{diag}\left({A_{t}}^{H}R_{0}A_{t}\right), \end{array}$$

where diag(·) denotes a vector of its diagonal elements, and $A_{t}$ is the matrix of transmit steering vectors, i.e., $A_{t}=\left[a_{t}\left(f_{b}\left(1\right)\right),\cdots ,a_{t}\left(N_{b}\right)\right]$.

A straightforward measure of the transmit beampattern mismatch is given by

(24) $$b_{v}=\left|b_{d}-\gamma b_{R}\right|,$$

where $\left|\cdot \right|$ denotes a matrix of absolute values of the matrix/vector entry. Here a parameter γ>0 is introduced to avoid amplitude ambiguity between expected transmit beampattern and real transmit beampattern.

In practice, the accuracy demanded is often different and this method can control the mismatch flexibly by adjusting the number of elements in $f_{b}$ and the relative weight in contrast to the sidelobe level.

### 2.6. Sidelobe Level Measure

In the receive end, there would be multiple space-time range compressors, each following a receive beamforming filter regarding a spatial direction. We intend to suppress sidelobe outputs in the range compressors, so we first define a group of angular frequencies regarding those range compressors or beamforming filters, by $f_{a}=\left[f_{a}\left(1\right),\cdots ,f_{a}\left(N_{a}\right)\right]$, where $N_{a}$ denotes the number of such a space-time range compressor in the receive end and $f_{a}\left(k\right)$ denotes the spatial direction regarding the *k*th range compressor. In practice, a peak of the transmit beampattern may need to deploy more than one such range compressor, depending on the width of the peak and system requirement.

Both auto- and cross-correlation sidelobes of angular waveforms regarding $f_{a}$ should be suppressed. Meanwhile, it has been shown in (20) that auto-correlation sidelobes are conjugate symmetric in the range shift dimension, so we need to suppress only one side of range sidelobes. For cross-correlation sidelobes, here we assume that the directional transmit beampattern to match has peaks with the same amplitude, and then from (21), there is also a conjugate symmetric property. Therefore, we define a measure of one-side sidelobe level to suppress by

(25) $${\mathrm{PSL}}_{a}=\max_{\begin{array}{c} k=1,2,\cdots ,N_{s}-1\\ f_{a}\left(m\right)\in f_{a} \end{array}}\left|{\bar{\rho }}_{k}\left(f_{a}\left(m\right)\right)\right|.$$

Although other spatial directions have low power allocation, returns in those directions may still have high power and then we should suppress them. It should be kept in mind that the interference of interest is one-way, i.e., only the interference to those spatial receive channels is of interest. In order to represent target returns from all other spatial direction, we define another group of angular frequencies by $f_{m}=\left[f_{m}\left(1\right),\cdots ,f_{m}\left(N_{m}\right)\right]$ to represent $N_{m}$ such attenuated spatial directions. To avoid duplicated sidelobes, we assume that $f_{m}$ and $f_{a}$ have no element in common. We define another PSL measure by

(26) $${\mathrm{PSL}}_{m}=\max_{\begin{array}{c} k=-N_{s},\cdots ,-1,1,\cdots ,N_{s}-1\\ f_{m}\left(m\right)\in f_{m},f_{a}\left(i\right)\in f_{a} \end{array}}\left|{\bar{\rho }}_{k}\left(f_{m}\left(m\right),f_{a}\left(i\right)\right)\right|.$$

### 2.7. Joint Waveform Optimization Criterion

Now we have defined two PSL measures, ${\mathrm{PSL}}_{a}$ and ${\mathrm{PSL}}_{m}$, as well as a transmit beampattern measure. They are combined to form the following waveform optimization criterion:

(27) $$\begin{array}{cc} \min_{\gamma ,\beta ,P} & \max\left[\alpha b_{v},{\mathrm{PSL}}_{a},{\mathrm{PSL}}_{m}\right]\\ s.t. & \sum_{i=1}^{N_{t}-1}\beta_{i}=D\\ \; & \beta_{i}\geq 1,i=1,\cdots ,N_{t}-1\\ \; & \gamma >0, \end{array}$$

where P=−jlog(S) denotes a matrix of phases of S, log(·) denotes the logarithm function, and α is a trade-off parameter between transmit beampattern matching and sidelobe level. In practice, the spacing of transmit antennas has a minimal limit, typically a half wavelength, so we impose a constraint over spacing for all antennas by $\beta_{i}\geq 1$. It is unnecessary to put a constraint on phase matrix P, whose elements have a period 2π. The optimization result of P would be shifted within the domain [0,2π].

Our optimization method can trade off between transmit beampattern matching performance and range sidelobe level. The trade-off parameter α is set offline, according to the interest of the designer. A high α would emphasize transmit beampattern performance and thus may sacrifice the sidelobe level. In practice, we do not agree with wasting too many degrees of freedom on transmit beampattern matching, because even though we match a beampattern accurately in theory, a real transmit array would be difficult to reproduce for array error and mutual coupling. Too accurate a transmit beampattern is not always necessary in some situations. Therefore, we advise relaxing transmit beampattern matching accuracy properly for a better PSL. The degree is key, and the weight should be tuned properly. In practice, sidelobes may be imposed by different weights for a desirable property and the extension is straightforward, so we do not show it explicitly here.

The problem in (27) is constrained, nonlinear, and NP-hard; the global minimum is difficult to reach and for such a problem, there are numerous optimization tools to use. Here we resort to the minimax algorithm based on the SQP, which is found to be efficient and robust [17].

## 3. Numerical Results

### 3.1. Optimization Configurations

The optimization is concerned with the background of a sparse MIMO transmit array. The transmit array has $N_{t}=12$ transmit elements, for which $N_{t}=128$ codes will be designed according to (27). The receive array deployed in the same direction has $N_{r}=12$ receive elements, all spaced by a half wavelength. As the SQP-based optimization algorithm is sensitive to the initial value, we run the optimization processing 20 times and select the best one as the final result for all the following numerical experiments.

**Figure 2:**
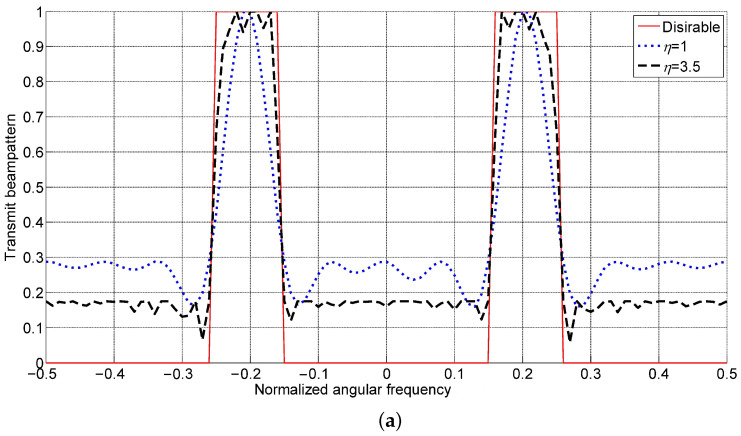

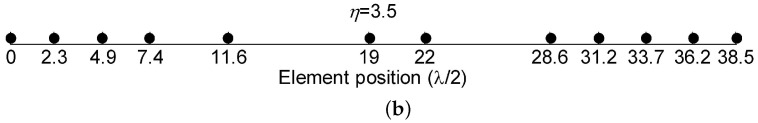
Results with P1: transmit beampatterns (**a**) and element locations (**b**).

For transmit beampattern matching, $f_{b}$ is quantized over [−0.5,0.5] Hz with interval 0.01 Hz, as shown in Figure 2. The directional transmit beampattern would have two peaks, located at $f_{c}=-0.2$ Hz and 0.2 Hz, so $f_{a}={\left[-0.2,0.2\right]}^{T}$ Hz. For each peak, we use four angular frequencies to represent target returns from other spatial directions and set $f_{m}={\left[-0.23,-0.2,-0.17,0,0.17,0.2,0.23\right]}^{T}$ Hz. The desirable transmit beampattern $b_{d}$ equals one for $f_{c}\in \left[-0.25,-0.15\right]\cup \left[0.15,0.25\right]$ and equals zero elsewhere.

Two simulations will be considered. The first aims at verification of the benefits induced by optimizing array spacing. The second aims at studying the impact of array aperture on optimization performance.

### 3.2. Benefits of Spacing Optimization

In [25], a waveform optimization algorithm without array spacing optimization is addressed, wherein there is also a trade-off parameter like α. To make a fair comparison with it, we set the trade-off parameter α=0.01 for both of them. Here the average element spacing is η=3.5, corresponding to a total aperture D=38.5 for the 12-element array. The transmit beampattern matching performance is shown in Figure 1, where the desirable transmit beampattern and that designed with η=1, corresponding to the half-wavelength and no-spacing-optimization case, are shown together.

Figure 2a indicates that the advantage of spacing optimization is obvious. The array spacing optimization gives rise to a better transmit matching performance than the method without spacing optimization. Meanwhile, additional degrees of freedom result in a lower sidelobe level of the transmit beampattern. Figure 2b shows the element positions after optimization, indicating that both the minimal spacing and the total aperture meet the prescribed settings.

**Figure 3:**
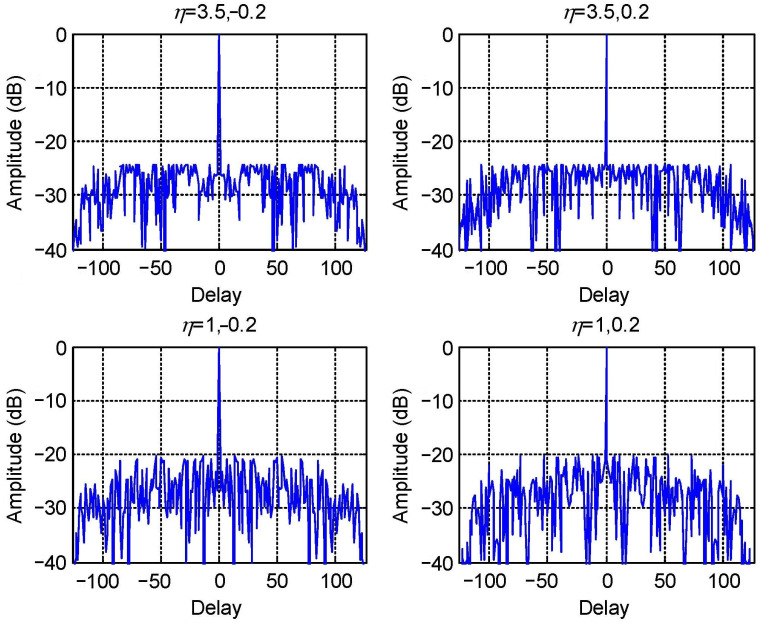
Auto-correlation sidelobes of angular waveforms in different configurations.

Auto-correlation sidelobes are shown in Figure 3, where the upper two figures are results with spacing optimization and the lower two are with the method without spacing optimization, both for two spatial directions corresponding to −0.2 Hz (left two) and 0.2 Hz (right two).

From Figure 3, the spacing optimization leads to a −24.4 dB auto-correlation ${\mathrm{PSL}}_{a}$, whereas no spacing optimization has only −20.2 dB. Therefore, additional degrees of freedom provide approximately a 4.2 dB reduction of ${\mathrm{PSL}}_{a}$.

**Figure 4:**
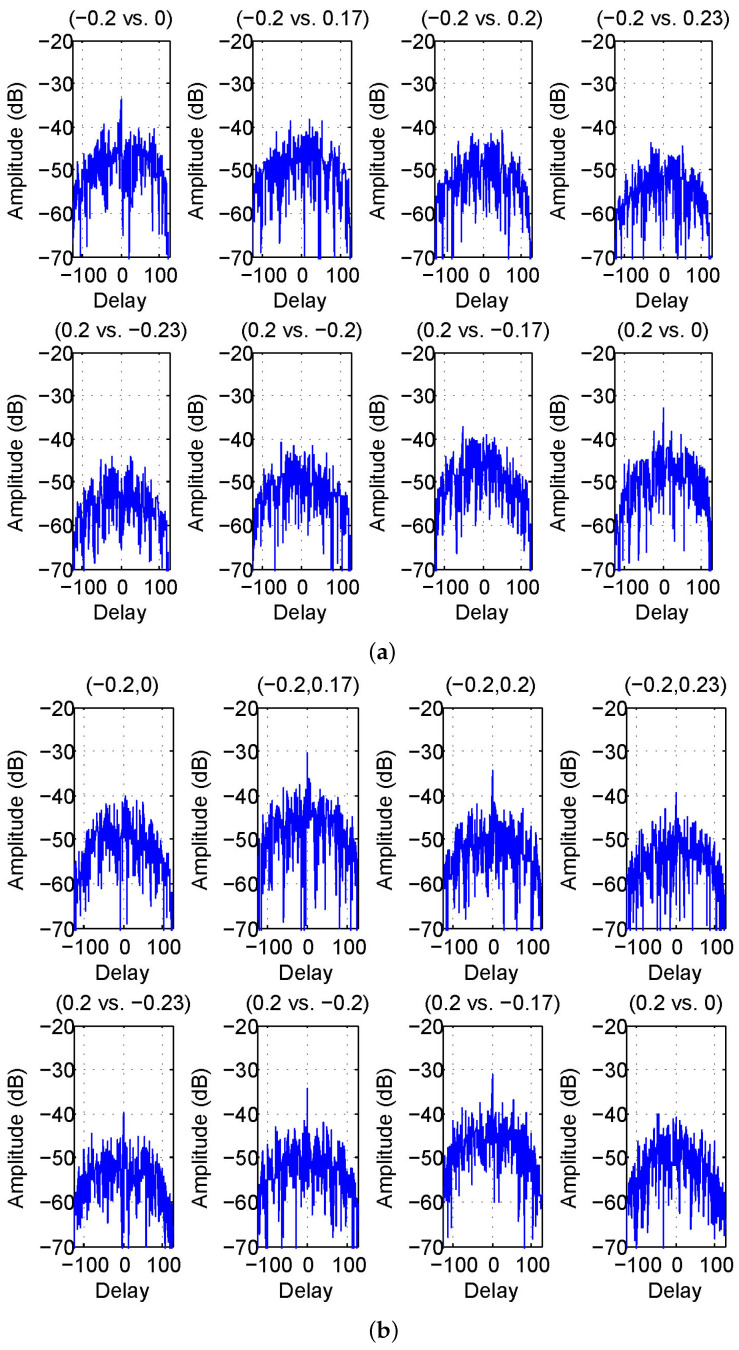
Mutual correlation sidelobes between angular waveforms for (**a**) our results and (**b**) another method in [25].

Two spatial receive channels would receive returns from directions other than $f_{a}$. To evaluate the effect, sidelobe outputs of returns from $f_{m}$ in the spatial receive channels are shown in Figure 4a,b, for our method and that in [25], respectively.

In Figure 4a,b, all sidelobe outputs are lower than −30 dB, much lower than auto-correlation sidelobes. Such a significant performance improvement is mainly caused by the receive beamforming operation, which has efficiently suppressed cross-correlation sidelobes of angular waveforms. Their difference is insignificant in this measure. For the 12-element receive array, the attenuation factors for the correlation pairs are shown in Table 1. The attenuation factor depends on the interval, i.e., a larger distance of two directions tends to have a lower attenuation factor. More attenuation factors regarding other correlation pairs can be computed through (2).

It can also be seen that those cross-correlation sidelobes are not as plain as auto-correlation sidelobes. That is because the waveform design criterion has equal weights on auto-correlation sidelobes, but auto-correlation sidelobes are more difficult to suppress through optimization than cross-correlation sidelobes. One can adjust the weight to meet specific demands. In [16], if the receive aperture is sufficiently large, one can even totally ignore cross-correlation sidelobes and focus on suppressing auto-correlation sidelobes for better performance.

### 3.3. Impact of Array Aperture

**Figure 5:**
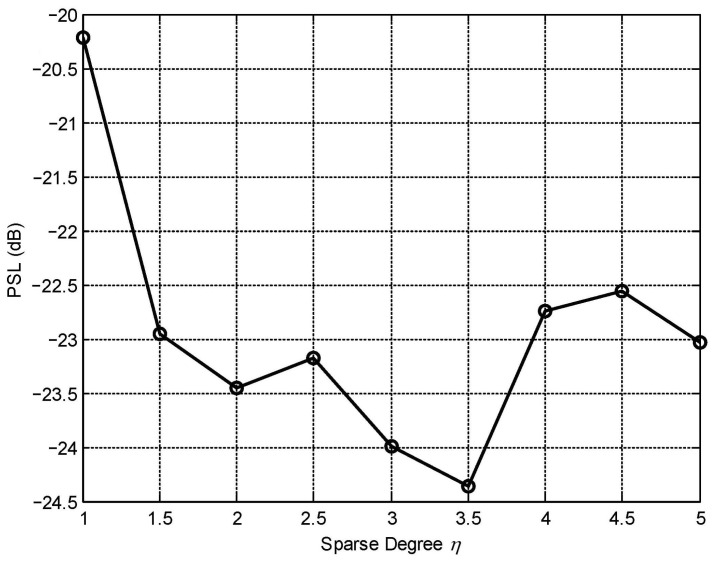
The PSL drop curve with the sparse degree.

Total transmit array aperture *D* determines the degrees of $\beta_{i}$ and thereby affects final performance. To study quantity, we run the optimization process nine times for η increasing from 1 to 5 with spacing 0.5 and show the PSL versus η in Figure 5. It can be seen that the increase in spacing can indeed lead to a lower PSL. However, there is a limit; the bonus will cease increasing after η reaches a point, approximately η=3.5 for our parameter settings.

**Figure 6:**
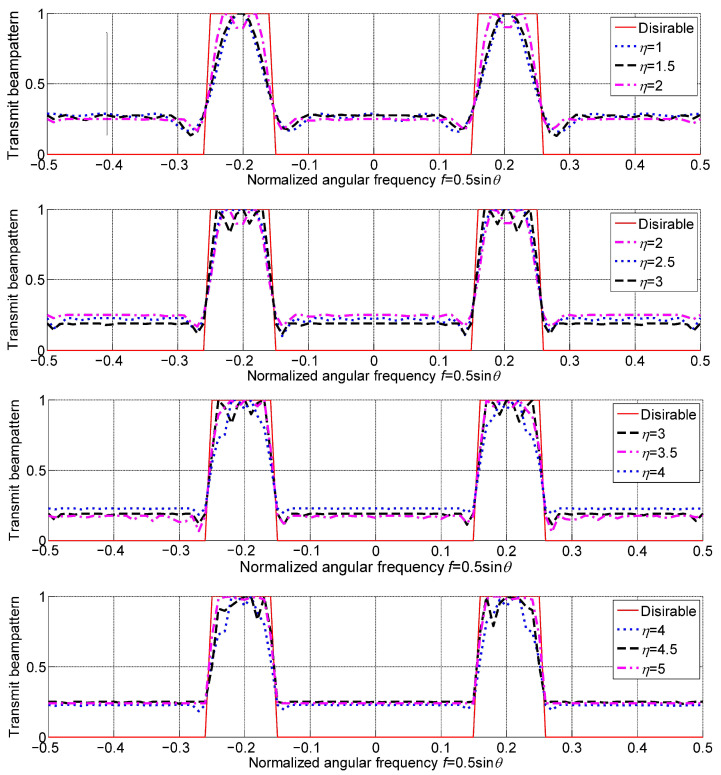
Transmit beampatterns for different sparse degrees.

The transmit beampattern matching performance would vary with the sparse degree η as well and the variation is shown in Figure 6, where different η are grouped into four figures to have a clear view. It can be found that the spacing optimization can enhance transmit beampattern matching performance, but like the sidelobe level, the matching performance will reach a limit. It will fluctuate after η reaches the limit.

In practice, different numbers of array elements may result in different turning points of η. For the case at hand, it is η=3.5, i.e., 3.5 times the half wavelength. It means that the degrees of freedom that can be extracted from spacing optimization have a limit. The final PSL is proportional to the transmit beampattern mismatch, so the total aperture has a similar effect on the PSL, which is not discussed here anymore.

## 4. Conclusions

Waveform design for colocated MIMO radar involves optimization for various variables and with numerous elements to suppress. The success lies greatly in the degrees of freedom available. In this paper, we study how to optimize transmit waveforms and array spacing of sparse colocated MIMO radar transmit array for a desirable transmit beampattern and a low sidelobe level output. We use array spacing optimization to exploit more degrees of freedom and incorporate the receive beamforming effect to make better use of existing degrees of freedom, so that both transmit beampattern matching performance and sidelobe level are improved, without grating lobes typically present in a sparse array. An attenuation factor is introduced to measure how much the receive beamforming would suppress mutual correlation sidelobes of angular waveforms. The factor is incorporated into our waveform design criteria and releases some degree of freedom that is originally allocated to suppress cross-correlation sidelobes of angular waveforms. Better use of degrees of freedom available reasonably brings a better performance output. The way to measure transmit beampattern matching performance and to quantize the angular frequency is also of interest to future waveform optimization. For simplicity, we consider a classical but simple receive beamforming algorithm, but this method can be generalized simply to more general quiescent receive beamforming algorithms, which may have different attenuations factors.

It is also revealed that the degrees of freedom that can be extracted from spacing optimization have a limit, i.e., average sparsity over about twice the wavelength may not reduce the PSL any more. Moreover, although spacing optimization enriches degrees of freedom and yields better optimization results, the reproduction in real applications depends heavily on the accuracy in controlling antenna locations. In practice, array spacing is applicable only for radar systems operating in a few carrier frequencies. In this case, one should extend our method to simultaneously suppress sidelobes over different frequencies. If a radar system operates in several frequencies, the performance may become worse for frequencies out of the optimization rule. An array spacing optimized for a carrier frequency may not be suitable for another carrier frequency. In order to improve the array manufacturing economy, some radar systems have a narrow operation bandwidth and this algorithm can make them work better. 

## Figures and Tables

**Table 1:** Attenuation factors for correlation pairs.

Correlation Pairs	(−0.2, 0)	(−0.2, 0.17)	(−0.2, 0.2)	(−0.2, 0.23)
Attenuation coefficient	0.13	0.09	0.05	0.04
